# Do patients consider computer-adaptive measures more appropriate than static questionnaires?

**DOI:** 10.1186/s41687-019-0096-3

**Published:** 2019-01-29

**Authors:** Eva-Maria Gamper, Caroline Martini, Morten Aagaard Petersen, Irene Virgolini, Bernhard Holzner, Johannes M. Giesinger

**Affiliations:** 1 Innsbruck Institute of Patient-centered Outcome Research (IIPCOR), Dr. Stumpf Strasse 56, 6020 Innsbruck, Austria; 2 Department of Psychiatry, Psychotherapy and Psychosomatics, Psychiatry II, Medical University of Innsbruck, Anichstrasse 35, 6020 Innsbruck, Austria; 3 The Research Unit, Department of Palliative Medicine, Bispebjerg Hospital, Bispebjerg Bakke 23, 2400 Copenhagen, Denmark; 4 Department of Nuclear Medicine, Medical University of Innsbruck, Anichstrasse 35, 6020 Innsbruck, Austria

**Keywords:** Patient-reported outcomes, Computer-adaptive tests, EORTC QLQ-C30, EORTC CAT, Mixed methods

## Abstract

**Objective:**

Computer-adaptive tests (CAT) use individualised sets of questions to assess patient-reported health states, whereas static (conventional) questionnaires present the same questions to all patients. CAT has been shown to increase measurement precision and reduce assessment length. In our study, we investigated if patients perceive CAT questions as more appropriate than static questionnaires, a claim that is frequently associated with CAT measures.

**Methods:**

We compared the static European Organisation for Research and Treatment of Cancer Quality of Life Questionnaire Core 30 (EORTC QLQ-C30) against its corresponding CAT measures focusing on two domains: Physical Functioning (PF) and Emotional Functioning (EF). Cancer patients completed the questionnaires and participated in a cognitive interview to assess how appropriate they perceive the QLQ-C30 and the CAT questions for their current health state.

**Results:**

Forty-four cancer patients (mean age = 54.6; 56.8% female) were assessed. For the PF domain, patients considered the CAT items more appropriate (*p* = 0.002) than the QLQ-C30 items (56.8% vs. 15.9%; 27.2% indifferent). For the EF domain, patients were in favour of the QLQ-C30 items (*p* < 0.001), with 54.5% considering the QLQ-C30, and 4.5% considering the CAT items to be more appropriate; 40.9% were indifferent. Most patients (*N* = 36) commented on the preference for the CAT (PF), mentioning better matching of the questions and the health state (38.6%) and better item wording (15.9%).

**Conclusion:**

For the PF domain the CAT measure better matched the score distribution in the patient sample than the QLQ-C30 PF scale and was consequently considered more appropriate by patients. For the EF domain, the CAT measure did not show better fit than the QLQ-C30 and hence no such preference in terms of appropriateness was observed.

## Background

In recent years, increasing efforts have been directed towards the incorporation of the patient’s perspective into medical care and research. Today, there is broad consensus on the importance of collecting so-called patient-reported outcomes (PROs), i.e. any report of the status of a patient’s health condition that comes directly from the patient, without interpretation of the patient’s response by a clinician or anyone else [1]. A large number of internationally used and well-validated PRO questionnaires are available for the assessment of various aspects of a patient’s health status such as pain, depression, anxiety, fatigue, or quality of life (QOL) [2].

PRO assessment still mostly relies on traditional questionnaires that present the same set of questions at every assessment to all patients and therefore are frequently referred to as being “static questionnaires”. The use of static PRO measures has shortcomings in terms of measurement precision and length as well as with regard to the comparability of scores across assessment instruments. Static questionnaires mostly are based on classical test theory and require a considerable number of items to cover the whole range of an assessed construct. Consequently, patients may be presented with a high proportion of items inappropriate for their current health state that, at the same time, do not increase measurement precision. For example, a patient who has difficulty taking a short walk will not provide additional information to the clinician or researcher when being asked questions about strenuous sports activities [3]. Moreover, answering such uninformative and inappropriate questions may be frustrating and affect patient compliance with questionnaire completion.

To overcome these limitations, a major focus of current PRO research is the development of computer-adaptive tests (CAT) [3–5] based on item response theory (IRT) measurement models. CAT represents a sophisticated form of assessing PROs more precisely by tailoring the questions to the health status of the individual patient. CAT requires an item bank containing a number of IRT-calibrated questions and an algorithm for selecting the most informative questions. A key item characteristic is the so-called item difficulty, a parameter that describes on which part of the measurement continuum an item provides the most information (i.e. has the highest measurement precision) [6]. The CAT algorithm is initiated with a start item and, based on the patient’s first response, calculates a first estimate of the level of the measured PRO. The algorithm then selects an item from the item bank whose item difficulty matches the patient’s construct level and administers this item next. This procedure is repeated until a predefined measurement precision has been reached or a maximum number of items have been asked. By using such tailored sets of items, measurement precision can be increased without increasing completion time.

A CAT version of one of the most widely used cancer-specific QOL questionnaires, the European Organisation for Research and Treatment of Cancer Quality of Life Questionnaire Core 30 (EORTC QLQ-C30) [7], has been developed by the EORTC Quality of Life Group (QLG) since 2005 within a series of projects and has been released recently [3, 8, 9]. These projects aimed at constructing item banks for computer-adaptive assessments of 14 out of the 15 functioning and symptom domains covered by the QLQ-C30 (excluding the global QOL scale). By now, several studies [9–11] have shown that the new CAT measures are superior to the original QLQ-C30 in terms of measurement precision. While expectations concerning measurement precision and unidimensionality of the item banks have been confirmed in these studies, another frequently claimed advantage of CAT measures has not been investigated so far, neither for the EORTC CAT measures, nor for other CAT measures: the expectation that patients perceive CAT items as more appropriate than items from static questionnaires, since they better match the individual health status.

To investigate this research question, we selected two key domains of the EORTC measurement system, namely physical functioning (PF) and emotional functioning (EF) and compared the static item sets of the QLQ-C30 against the computer-adaptive measures [3, 8, 9, 12] with regard to patients’ perceptions of the appropriateness of the administered questions.

We chose these two domains, as score distributions in the majority of cancer patient groups [13] and the comparison of the measurement precision of the QLQ-C30 and the new CAT item banks [9, 14] indicated that for PF the CAT measures may be superior, in particular in patients with high levels of functioning, whereas for EF no substantial benefit could be expected in well-performing patients.

Therefore, we hypothesised that patients with high levels of functioning would perceive the CAT items to be more appropriate than the QLQ-C30 items for the PF domain, but there would be no difference concerning appropriateness between the two measures for the EF domain.

## Patients and methods

### Sample and procedure

The study was conducted at a Nuclear Medicine Department. At this department, routine electronic PRO monitoring has been in place since 2011 using the software CHES [15]. The routine assessment comprises the QLQ-C30 and the respective disease specific modules that are completed at the beginning of an inpatient stay. The majority of patients admitted at the department are treated for a diagnosis of thyroid cancer or a neuroendocrine tumour. We approached patients consecutively for study participation in line with the following inclusion criteria:

- Diagnosis of thyroid cancer or neuroendocrine tumour
- Sufficient command of German
- No obvious cognitive impairment
- Age between 18 and 80 years
- Participation in routine PRO monitoring

A trained interviewer informed patients about the study objective and procedure. Patients who agreed to participate were assessed with the QLQ-C30 as part of the routine PRO monitoring and completed in addition the EORTC CAT measures for PF and EF. The electronic assessment procedure did not reveal whether an item was part of the QLQ-C30 or of the CAT measure. To elicit the patient’s perspective on the appropriateness of questions, we conducted a cognitive interview within less than one hour after questionnaire completion.

We planned to recruit a minimum number of 30 patients and then continued recruitment until thematic saturation in the cognitive interview had been reached, i.e. no new issues appeared during five subsequent patient interviews. Sociodemographic and clinical data were gathered from the medical charts.

### Assessment instruments

#### EORTC QLQ-C30

The EORTC QLQ-C30 [7] is a widely used cancer-specific QOL questionnaire that can be supplemented with disease-specific modules. It consists of 30 items forming 5 multi-item functioning scales (physical, role, emotional, cognitive, social), 3 multi-item symptom scales (fatigue, nausea/vomiting, pain), 6 single-item symptom scales (dyspnoea, insomnia, appetite loss, constipation, diarrhoea, financial difficulties) and a global QOL scale. Scale scores are derived from summing up item scores followed by linear transformation to a metric from 0 to 100. For the functioning scales high scores indicate good health, whereas for the symptom scales high scores indicate poor health.

#### EORTC CAT measures for physical and emotional functioning

The item bank for the EORTC PF CAT comprises 31 items [3, 8] and the item bank for the EORTC EF CAT 24 items [9, 12]. All EORTC CAT item banks are designed to measure the same constructs as the corresponding scale of the QLQ-C30 and include all items of this instrument. Scores obtained from EORTC CAT measures are fully backward compatible to scores derived from the QLQ-C30. Backward-compatibility was a key aspect of the development process of the EORTC CAT measures and was ensured for item content and conceptualisation [3, 12] and for dimensionality [8, 9].

In our study, the number of items to be administered in the CAT was set to 5 items for PF and 4 items for EF to equal the number of items in the QLQ-C30 for both domains. The starting items of the CAT measures were according to the expected mean scores of PF and EF in the patient sample. Expected values were estimated using the QLQ-C30 data collected at the department previously within the routine PRO monitoring (mean PF 81 points, mean EF 67 points).

#### Cognitive interview

We developed a semi-structured interview to assess patients’ perceptions of the appropriateness of the QLQ-C30 and CAT items based on the cognitive interviewing technique (think-aloud sessions) [16] and pilot-tested it for clarity and feasibility in patients at the department. The interview included an explanation of the constructs of PF and EF as assessed within the EORTC measurement system. During the interview, we asked patients to choose which of the two sets of items (QLQ-C30 vs CAT items on PF and EF) they considered to have been more appropriate for their condition. The two item sets were presented as separate lists that did not show whether it were CAT or static items. The interviewer also encouraged patients to give reasons for decisions, thoughts and comments on individual items.

### Data analysis

Sample characteristics are provided as means, standard deviations, ranges and absolute and relative frequencies. Item exposure of the CAT items (i.e. the frequency of administration of individual items from the item bank) is given as absolute frequencies. In addition, we provide descriptive statistics for the score distribution of the PF and EF QLQ-C30 scales. The comparison of patients’ appropriateness rating was done using a sign test, a non-parametric test for dependent variables. The impact of sociodemographic characteristics on appropriateness ratings was assessed using the Kruskal-Wallis test for metric and ordinal variables and a Chi-Squared test for nominal variables as appropriate. Furthermore, we investigated whether patients had rated the question set that was psychometrically more informative. To do so, responses were IRT-scored and the information of the QLQ-C30 static question sets and the CAT question sets, respectively, were determined [8, 9]. We calculated the difference in information between the two question sets, and split the sample at the median into a ‘small difference in information group’ and a ‘large difference in information group’ and compared the appropriateness ratings for CAT or the QLQ-C30 respectively in these two groups. The qualitative interview data were categorised by two authors using categories that were set up based on a sample of the interview data. For each domain, we distinguished positive and negative comments on CAT and the QLQ-C30. Results are provided as absolute frequencies per category and with individual patient quotes.

## Results

### Sample characteristics

In total, 65 eligible patients were approached for study participation. Fourteen patients did not participate due to the following reasons: burden of completing questionnaires (*n* = 8), not understanding the study purpose (*n* = 3), technical problems with electronic data capture (*n* = 2) and concerns on the use of the questionnaire data (*n* = 1). Of the remaining 51 patients, 7 patients were included in the pilot testing of the cognitive interview and 44 in the main study. Participants in the main study had a mean age of 54.6 years and were mostly women (56.8%). The majority of patients were suffering from thyroid cancer (79.5%). Most common comorbidities according to the Charlson Comorbidity Index [17] were myocardial infarction (6.8%) and previous cancer (4.5%). The vast majority of patients (90.9%) had previous tumour resection (median time since surgery was 8.5 months). Most common medication was thyroid medication (84.1%), antihypertensive medication (36.4%) and psychopharmacological medication (29.5%). See Table 1 for further details on patient characteristics.

**Table 1:** Sample characteristics (*N* = 44)

	Mean (*SD*)	Range
Age (years)	54.6 (14.7)	26–80
	N	%
Sex		
Women	25	56.8
Men	19	43.2
Marital status		
In Relationship	31	70.5
Single	13	29.5
Education		
Compulsory school (≤9 years)	3	6.8
Apprenticeship/prof. School (9–12 years)	25	56.8
A-levels (12–13 years)	9	20.5
University degree (≥15 years)	4	9.1
Other	3	6.8
Employment		
Full-time	8	18.2
Part-time	7	15.9
Homemaker	3	6.8
Retired	16	36.4
Self-employed	4	9.1
Other	6	13.6
Diagnosis		
Thyroid cancer	35	79.5
Neuroendocrine tumour	9	20.5
Previous tumor resection		
Yes	40	90.9
No	4	9.1
Current treatment phase		
No treatment/follow-up	32	72.7
Nuclear therapy	12	27.3
Most common medications		
Thyroid hormones	37	84.1
Antihypertensives	16	36.4
Psychopharmacological medication	13	29.5
Most common comorbidities		
Myocardial infarction	3	6.8
Previous cancers	2	4.5
Previous routine PRO assessments		
None	19	43.2
1	13	29.5
2	5	11.4
3 or more	7	15.9

### Score distribution and item exposure

For the QLQ-C30 PF scale the median score was 87 points (*M* = 79.9, *SD* = 21.0). A ceiling effect of 22.7% (i.e. total score of 100) and no floor effect (i.e. total score of zero) were observed. For the QLQ-C30 EF scale the median score was 67 points (*M* = 63.4, *SD* = 27.2) and a ceiling effect of 15.9% and a floor effect of 4.5% were observed. Frequency of patients scoring the top range between 90 and 100 score points was 43.2% for the PF domain and 22.7% for the EF domain.

In total, 220 CAT items were administered for PF (5 items per patient) and 176 for EF (4 items per patient). Analysis of item exposure in the CAT assessments showed that for PF 9.1% (20 out of 220) of all administered items were items from the QLQ-C30. The PF CAT showed a ceiling effect of 6.8% and no floor effect. For EF 27.8% (49 out of 176) of all items administered in the CAT were items from the QLQ-C30. The EF CAT showed a ceiling effect of 15.9% and a floor effect of 2.3%. Details on item exposure are given in Table 2.

**Table 2:** Item exposure for the Physical Functioning and Emotional Functioning CAT

Item text	*N*
Physical Functioning	
Do you have any trouble carrying a heavy bag upstairs?	44
Do you have any trouble taking a long walk carrying a heavy pack on your back (e.g. a filled rucksack)?	33
Do you have any trouble running a short distance, such as to catch the bus?	31
Do you have any trouble running 100 m?	24
Do you have any trouble running fast?	21
Do you have any trouble hiking 3 km on uneven surfaces?	18
*Do you have any trouble doing strenuous activities, like carrying a heavy shopping bag or a suitcase?	10
Do you have any trouble carrying something in both hands (e.g. shopping bags) while climbing a flight of stairs?	10
Do you have any trouble walking for 30 min.?	8
*Do you have any trouble taking a long walk?	7
Do you have any trouble walking 100 m?	5
Do you have any trouble walking outdoors on flat ground?	4
*Do you have any trouble taking a short walk outside of the house?	2
Do you need help undressing?	2
*Do you need help with eating, dressing, washing yourself or using the toilet?	1
Emotional Functioning	
Have you felt miserable?	44
Have you felt sad?	27
Have you felt that nothing could cheer you up?	20
*Did you feel depressed?	17
*Did you feel tense?	16
*Did you worry?	16
Have you felt desperate?	11
Have you felt discouraged?	10
Have you felt helpless?	9
Have you felt like giving up?	5
Have you been afraid of losing control?	1

Note. * = Items from the QLQ-C30; total number of items administered: EF 4 × 44 = 176, PF 5 × 44 = 220; the table only shows items that were administered at least once

### Appropriateness of the QLQ-C30 and the CAT measures

For PF most patients (56.8%) considered the CAT items to be more appropriate for them than the QLQ-C30 items (*p* = 0.002), whereas for EF most patients (54.5%) rated the QLQ-C30 items to be more appropriate than the CAT items (*p* < 0.001). For PF 27.2% and for EF 40.9% of patients were indifferent. For further details, see Table 3.

**Table 3:** Appropriateness rating CAT vs. QLQ-C30 questions

	Physical Functioning				Emotional Functioning			
	CAT	QLQ-C30			CAT	QLQ-C30		
Information*	28.76	10.96			12.04	6.83		
Median of difference in information	18.59				4.45			
Preferences	CAT	indifferent	QLQ-C30		CAT	indifferent	QLQ-C30	
Overall	56.8% *n* = 25	27.2% *n* = 12	15.9% *n* = 7	*p* = 0.002	4.5% *n* = 2	40.9% *n* = 18	54.5%*n* = 24	*p* < 0.001
Large difference in information group	68.2% *n* = 15	22.7% *n* = 5	9.1% *n* = 2		9.1% *n* = 2	22.7% *n* = 5	68.2% *n* = 15	
Small difference in information group	45.5% *n* = 10	31.8% *n* = 7	22.7% *n* = 5		0	59.1% *n* = 13	40.9% *n* = 9	

Note. * = Mean test information values (i.e. measurement precision) derived from IRT models used for item calibration [9, 10]. Please note that there are no absolute values for test information

There was no significant association of age with the appropriateness ratings on the PF domain (*p* = 0.194). For EF there was a significant association with age (*p* = 0.038), indicating that patients who were indifferent were older (*M* = 61.2, *SD* = 14.6) and patients who rated the QLQ-C30 to be more appropriate were younger (*M* = 50.0, *SD* = 13.9). No significant association was observed for sex (PF: *p* = 0.715, EF: *p* = 0.886) and education (PF: *p* = 0.515, EF: *p* = 0.726).

From a psychometric perspective, CAT provided more information than the QLQ-C30 for all participants (see Table 3). Splitting the sample at the median into a “small difference in information group” and a “large difference in information group” and comparing appropriateness ratings for CAT vs. the QLQ-C30 in these groups revealed that for PF a small difference in information was associated with only small differences in appropriateness ratings (45.5% CAT, 31.8% indifferent, 22.7% QLQ-C30) and a large difference in information was associated with a clearly higher subjective appropriateness of CAT (68.2% CAT, 22.7% indifferent, 9.1% QLQ-C30). For EF it could be shown that when the difference in information was small, the majority of patients (59.1%) were indifferent and when the difference in information was large, the majority (68.2%) rated the QLQ-C30 to be more appropriate (see Table 3).

### Qualitative interview results

The cognitive interview data was categorised into positive and negative aspects of the static and of the computer-adaptive measures separately for the PF and for the EF domain. Thirty-six patients provided comments concerning the PF domain and 8 patients commented on the EF domain. For the PF domain, positive aspects of the CAT items mostly related to a better match with the current physical state that required questions on demanding activities (17 patients). In addition, the CAT PF items were considered to be better worded and easier to answer (7 patients). The latter was stated for the QLQ-C30 items by 6 patients. For the EF domain, 3 patients mentioned the CAT items to be too extreme for their current emotional condition, whereas two patients considered them to be highly appropriate. Three patients liked the wording of the QLQ-C30 better. Further details are given in Table 4.

**Table 4:** Results from cognitive interview think-aloud sessions

		QLQ-C30	CAT
Physical Functioning	Positive	better worded and easier to answer (*n* = 6):	matches the current physical state better (*n* = 17):
		“questions are worded more generally”, “more efficient”, “questions are clearly formulated and can be answered without any problems”, “more pleasing”	“aiming at people who are agile and do a lot of sports”, “described activities are more physically demanding”
			better worded and easier to answer (*n* = 7):
			“you can answer more accurately”, “concrete examples including durationare very helpful”
	Negative	not suitable for current physical state (*n* = 4): “questions for people with limited mobility”, “better for people who are untrained or out of condition”	not suitable for current physical state (*n* = 3):
			“have not done this recently due to less physical activity”
		difficult to answer (*n* = 3): “questions are not formulated clearly”, “questions mean different things to different people”	difficult to answer (*n* = 1):
			“described activities are very specific”
Emotional Functioning	Positive	better worded and easier to answer (*n* = 3):	highly appropriate and more precise (*n* = 2)
		“questions are worded more generally”, “exact wording”, “more pleasing”	
	Negative	–	too extreme for current emotional condition: “dramatizes”, “sounds like being suicidal” (*n* = 3)

## Discussion

Our study investigated cancer patients’ perceptions of the appropriateness of questions on PF and EF, comparing the questions from the static EORTC QLQ-C30 against the corresponding CAT measures. We found that patients considered the CAT PF to ask more appropriate questions than the PF items in the QLQ-C30, whereas the opposite was observed for the EF domain, i.e. higher subjective appropriateness of QLQ-C30 items. These effects were especially pronounced in those patients for whom, from a psychometric perspective, the information gained through CAT was large. These findings are well in line with our hypothesis for the PF domain, but not for the EF domain, where we expected more balanced appropriateness ratings of the static and the computer-adaptive questions.

As expected, the QLQ-C30 score distribution was more skewed in the PF domain than in the EF domain. This score distribution (i.e. the non-normal distribution for PF due to ceiling effects) indicates a mismatch of the QLQ-C30 items and the investigated population. This mismatch was also found in the analysis of item exposure where in the CAT assessment of PF the five QLQ-C30 items represented only 9% of all individual items asked. For the EF domain, the score distribution indicated a better spread across the measurement range and therefore a better match of the QLQ-C30 items and the population. In line with this, the EF CAT algorithm selected to a larger degree QLQ-C30 items (28% of the administered items) which indicates that the QLQ-C30 items were among the most informative in this patient group.

For PF the QLQ-C30 asks, for example, about needing assistance for self-care while patients in our sample were more concerned with whether or not being able to perform strenuous sports activities. For the assessment of EF on the other hand, in this patient group the QLQ-C30 items were measuring very well around the sample mean. Apparently in this case the QLQ-C30 static items set for EF often was considered to be more appropriate than CAT EF items. This unexpected finding may have been a result of the fact that the QLQ-C30 items cover core aspects of EF with easy understandably and very familiar expressions for emotional distress. Further, the CAT items mainly concern more severe emotional states than the QLQ-C30, i.e. the far majority of the CAT items are primarily relevant for patients with poorer EF than most patients in the current sample. Had the sample included more patients with low EF scores the appropriateness ratings might have been different. Furthermore, the results on EF suggest that while patients may like simpler items better, particularly for an abstract and complex construct as EF, the simple items may not necessarily be the most informative ones from a psychometric perspective. Hence, it may sometimes be necessary to ask more demanding and potentially semantically distressing questions to obtain the desired knowledge, even though patients may prefer simpler ones.

These differences in preference for PF and EF clearly illustrates that the advantage of CAT and its ability to adapt to the individual depends crucially on that relevant items are available in the item bank. For the current patient sample the PF item bank included several items more relevant than the QLQ-C30, hence the CAT was considered more appropriate, while this was not the case for EF item bank, which may lack items particularly relevant for patients with few emotional problems [9].

Results from the cognitive interviews for PF also emphasise the importance of asking items whose difficulty levels match the patient’s PF level. Furthermore, qualitative results did not indicate relevant differences between QLQ-C30 and CAT PF items with regard to how clearly they were formulated and how easy they were to answer. For the EF domain, patients provided very few comments, which relates to the fact that about 40% were indifferent concerning appropriateness of administered questions for this domain. In addition, it may also reflect that EF is a complex construct and therefore it is intellectually challenging to justify a decision on appropriateness and verbalise arguments.

A limitation of our study is that not all patients were presented the QLQ-C30 items for the first time, as some already had participated in the routine PRO monitoring at the department at previous admissions. This could have resulted in a preference for QLQ-C30 items, since they were familiar to some patients. However, this would affect the PF as well as the EF domain.

While the sample size is not large, it was sufficient to reach thematic saturation in the qualitative part and to demonstrate significant differences with regard to appropriateness ratings. Analysis of the impact of patient characteristics on preference had limited statistical power though. Cognitive interviewing revealed that while patients easily could state a subjective preference for a certain item set, it was very difficult for them to provide explicit justification for their decision. This somewhat limited the fruitfulness of the qualitative part of the interview. It is also important to note, that the CAT has also benefited from a start item that we selected based on previous data collected at the department. Such tailoring is not possible for traditional questionnaires like the QLQ-C30, but for static short-forms based on item banks. The latter can be adapted a priori based on assumptions on score distribution in the target population. This type of IRT-based static questionnaires has been shown to have a measurement precision close to that of CAT measurements [18]and may therefore also be considered to be more appropriate than traditional questionnaires by patients.

## Conclusion

In summary, our results support the assumption that questions with a difficulty level that corresponds to the patient’s level of functioning are not only more informative and precise from a psychometric perspective, as demonstrated previously [10, 19, 20], but also perceived as more appropriate by the patient him/herself. This would provide another strong argument for the use of computer-adaptive measures. Larger follow-up studies on the issue investigating other HRQOL domains and other CAT measures are warranted.

## Abbreviations

EF: Emotional Functioning
EORTC: European Organisation for Research and Treatment of Cancer
IRT: Item response theory
PF: Physical Functioning
PROs: Patient-reported outcomes
QLG: Quality of Life Group
QLQ-C30: Quality of Life Questionnaire Core 30
QOL: Quality of life
