# Correction to: do patients consider computer-adaptive measures more appropriate than static questionnaires?

**DOI:** 10.1186/s41687-019-0103-8

**Published:** 2019-02-19

**Authors:** Eva-Maria Gamper, Caroline Martini, Morten Aagaard Petersen, Irene Virgolini, Bernhard Holzner, Johannes M. Giesinger

**Affiliations:** 1 Innsbruck Institute of Patient-centered Outcome Research (IIPCOR), Dr. Stumpf Strasse 56, 6020 Innsbruck, Austria; 2 Department of Psychiatry, Psychotherapy and Psychosomatics, Psychiatry II, Medical University of Innsbruck, Anichstrasse 35, 6020 Innsbruck, Austria; 3 The Research Unit, Department of Palliative Medicine, Bispebjerg Hospital, Bispebjerg Bakke 23, 2400 Copenhagen, Denmark; 4 Department of Nuclear Medicine, Medical University of Innsbruck, Anichstrasse 35, 6020 Innsbruck, Austria

**Correction to**: **Journal of Patient-Reported Outcomes (2019) 3:7**

**https://doi.org/10.1186/s41687-019-0096-3**

Following publication of the original article [1], the authors reported three of their given name have been erroneously tagged as their family names. The correct names are: give name Caroline family name Martini, give name Irene family name Virgolini, give name Bernhard family name Holzner.

It has been corrected in the original article as well.

The publisher apologizes for any inconvenience caused by this error.
